# Wants to Know You Know

**Published:** 1885-12

**Authors:** 


### WANTS TO KNOW, YOU KNOW.

A writer in that luminary which sheds so much obscure brilliancy and grinds out so much questionable wisdom, "*The Brief*," asks for information on what he calls "a point of great medico-legal importance," as follows:

"Mr. A. had mumps; they fell; right testicle perished away; his wife had, afterwards, three children, two girls and one boy.

Mr. A. thinks *they should all have been girls*, and that he is entitled to a divorce, and has consulted me. I am of opinion *he is*, but concluded to ask somebody who knows before committing myself."

Very wise conclusion, Doctor.

On reading the above to Dr. Merryman, who had dropped in, he remarked. "I feel like exclaiming as Bill Nye did, when Ah Sin got the three bowers "which the same had been dealt unto him"—"Can such things be?" Dr. M. further remarked: "I think Mr. A. would have done better if he had consulted his mule; in that event there would have been no danger of separating man and wife—ignorant and credulous though the man be;—of bastardizing innocent children, nor of sending an unoffending wife to perdition."

Dr. M. says he came near exclaiming: "the infernal donkey," but, said he: "What has that long (eared) suffering animal done to me that he should be subjected to such a comparison? Can't somebody sit down on the Brief? Or *squib-nobble* some of its correspondents?"

"What is squib-nobble, Doctor?" we asked.

"It is an operation known in the vernacular as "boring for simples." It is derived from the *Sanscrit*, *squibob*, to add to, and *nobbob*, the excrementitious product of the owl or bat, and consists of filling the vacancy usually found on opening the skull of certain persons, with putty; or better still, especially if there be any substance present which does duty as brain matter, of replacing it with a little very fine *guano*. This has been found to be a great improvement on the operation, and also on the mental condition of certain persons affected with the *cocœthes scribendi*."

We accused the Doctor of coining the word, and he said he had as much right to do so as any other learned professor. He cited Prof. D. B. St. John Roosa, who uses *presby*, "an old man," and *kousis*, "can hear," to express senile *deafness;*—and said he,—"it strikes *me* what the D. B. Professor wants in that case, is a couple or so of words which, put together, mean an old man or an old woman who *can't* hear, *eh*?"

For fear this may not be appreciated, we will explain that Merryman is a wag, and always "full of his jokes."

